# Comparing outcomes of operative management of intestinal obstruction due to gallstone ileus using NSQIP database

**DOI:** 10.1016/j.sipas.2023.100203

**Published:** 2023-07-17

**Authors:** Varun Rao, Genaro DeLeon, Timothy Becker, Benjamin Duggan, Kevin Y. Pei

**Affiliations:** a Indiana University School of Medicine, 340W 10th St, Indianapolis, IN 46202, USA; b Parkview Health Graduate Medical Education, 2200 Randallia Dr., Fort Wayne, IN 46805, USA

**Keywords:** ACS-NSQIP, Cholecystectomy, Gallstone ileus, Outcomes, General surgery

## Abstract

**Introduction:**

Gallstone ileus is an uncommon etiology of intestinal obstruction, although many cases require surgical repair. There is no consensus regarding the necessity of concomitant cholecystectomy. (CCY) at the time of index surgery. This study aimed to evaluate the outcomes of gallstone ileus in patients with and without CCY.

**Methods:**

Using the ACS NSQIP database from 2005 to 2019, we included patients who underwent surgical management of gallstone ileus (enterolithotomy) with or without CCY. The primary outcomes of interest were surgical site infection (SSI) and 30-day mortality. Additional outcomes of interest included readmissions related to the procedure, length of hospital stay (LOS), return to the operating room, and sepsis. Demographics were evaluated using univariate analysis, whereas outcomes of interest were analyzed using multivariate logistic regression.

**Results:**

A total of 825 cases of gallstone ileus were identified among 118 patients who underwent cholecystectomy. Patient characteristics were similar between the groups. No concomitant cholecystectomy was associated with a longer hospital stay (8 days vs. five days, *p*<0.01) and tended to be more likely to return to the operating room (45 cases vs. 4 cases, *p* = 0.08), but this was not statistically significant. No concomitant CCY was associated with increased SSI rates, readmissions related to the procedure, 30-day mortality, or sepsis.

**Conclusion:**

Surgical management of gallstone ileus with or without CCY has similar short-term postoperative outcomes.

## Highlights

• Currently, there is no consensus in terms of necessity of concomitant cholecystectomy at time of enterolithotomy.
• Comparable short-term postoperative outcomes were observed in the surgical management of gallstone ileus with or without cholecystectomy.
• Our study revealed that excluding concomitant cholecystectomy was linked to an extended hospital stay and a potential increase in the likelihood of requiring a subsequent operation.

## Introduction

Gallstone ileus is an uncommon etiology of intestinal obstruction, although many cases require surgical repair. This complication of cholelithiasis accounts for only 1–4% of mechanical bowel obstruction, but this increases to 25% of bowel obstruction in the elderly with high morbidity and mortality [1], [2], [3]. Surgical management of gallstone ileus varies depending on patient comorbidities, age, surgeon experience, and intraoperative anatomical difficulties [2,[4], [5], [6]]. There is no consensus regarding the necessity of cholecystectomy (CCY) at the time of the index operation. The current literature is split regarding surgical decision-making: choosing between enterolithotomy alone or a one-stage procedure in which enterotomy, CCY, and fistula resection are completed concomitantly [5,6]. A small retrospective study from 1997 reported a significant reduction in the overall mortality rate with the one-stage procedure compared with enterolithotomy alone [7]. However, more recent studies have proposed only offering a one-stage procedure to stabilized or low-risk patients, while enterolithotomy alone remains the best option [8,9]. Other studies have shown increased mortality associated with the one-stage procedure, recommending that the procedure should only be considered in particular patients on an elective basis [10,11]. This study aimed to provide an updated evaluation of the outcomes of gallstone ileus repair with or without CCY using a national surgical database.

## Methods

### Data source

This study utilized retrospective data from the American College of Surgeons National Surgical Quality Improvement Program (ACS NSQIP). This program is a database that collects preoperative risk factors and 30-day postoperative patient outcomes data from multiple institutions. The patients included in the NSQIP were risk-adjusted and case-mix-adjusted to enable comparison. The study protocol was exempted from institutional review board approval.

### Study design

This study had a retrospective cohort design using the ACS-NSQIP database. Patients were queried based on the International Classification of Diseases (ICD) and current procedural terminology (CPT). ICD-9 code 560.3, and ICD-10 code K56.3 for the diagnosis of gallstone ileus, were used. CPT codes 47562–47564 for laparoscopic procedures on the biliary tract and 47600–47620 for cholecystectomies were also used. Patients with other ICD or CPT codes were excluded if they did not fit the aforementioned codes.

The patients were analyzed for specific demographic and comorbid preoperative variables. The primary outcomes of interest were surgical site infection (SSI) and 30-day mortality. The secondary outcomes of interest included readmissions related to the procedure, length of hospital stay, return to the operating room, and sepsis.

### Statistical analysis

**Fig. 1:**
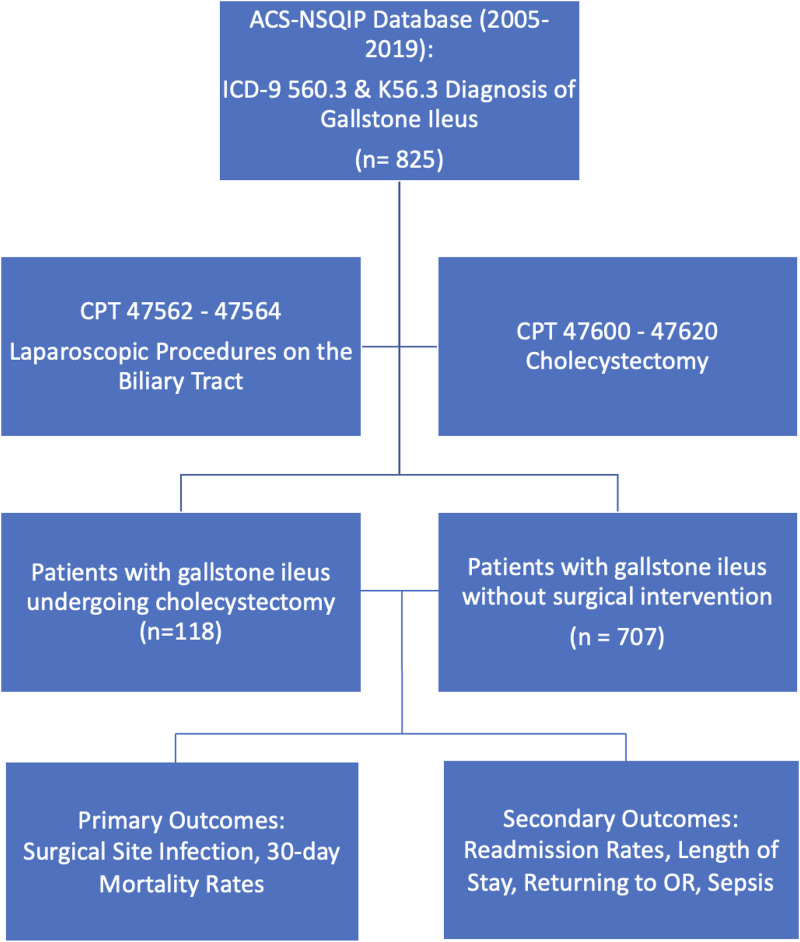
Methodology and data analysis structure.

Demographic and preoperative characteristics were evaluated using the Wilcoxon rank-sum test for continuous variables and Fisher's exact test for categorical variables. Outcomes of interest were analyzed using multivariate logistic regression while adjusting for patient characteristics. P-values and confidence intervals were set to 0.05. The analysis was performed using R version 3.6.1. The methodology is summarized in Fig. 1.

## Results

### Demographics, preoperative characteristics, comorbid conditions

A total of 825 patients with gallstone ileus repair underwent surgical intervention, with 118 of these patients also receiving concurrent CCY. The study included 68.2% female and 31.8% male patients. Among all the surgeries included in the study, 54.3% were classified as emergent.

Patients who underwent gallstone ileus repair alone had an average age of 50 years, whereas those who underwent CCY had an average age of 38 years (*p*<0.001). Certain preoperative variables in the non-CCY group without CCY differed significantly from those in the CCY group. The white blood cell count (11.8 vs. 8.8, *p*<0.01) and alkaline phosphatase levels (88 vs. 76, *p*<0.01) were higher in the no CCY group. Additionally, the no-CCY group had a significantly higher number of emergent operations (412 vs. 36, *p*<0.01), and the duration of surgery was significantly higher in this group (62 min vs. 96 min, *p*<0.01).

Table 1 shows demographic characteristics, comorbid variables, preoperative variables, and intraoperative variables for patients who underwent gallstone ileus repair alone versus those who underwent gallstone ileus repair with CCY.

**Table 1:** Comparison of preoperative and intraoperative characteristics in patients undergoing gallstone ileus repair with or without CCY.

Variable	No CCY (*n* = 707)	CCY (*n* = 118)	*P*
Demographics Age, median (IQR) Sex Female (%) Male (%) Body Mass Index, median (IQR)	50 (41–59) 483 (68.3%) 224 (31.7%) 27 (23–34)	38 (26–49) 80 (67.8%) 38 (32.2%) 28 (24–31)	<0.001 >0.9 0.7
Comorbid Variables (%) Diabetes Mellitus Hypertension Tobacco Use Congestive Heart Failure Chronic Obstructive Pulmonary Disease Bleeding Disorders Dialysis	170 (24.0%) 434 (61.4%) 67 (9.5%) 9 (1.3%) 28 (4.0%) 76 (10.8%) 5 (0.7%)	19 (16.1%) 59 (50.0%) 15 (12.7%) 0 (0.0%) 2 (2.0%) 5 (4.2%) 0 (0.0%)	0.3 0.02 0.3 0.3 0.3 0.03 >0.9
Preoperative Physiologic Variables ASA Classification ≥ 3 (%) Preoperative White Blood Cell Count, median (IQR) Preoperative bilirubin, median (IQR) Preoperative serum glutamic oxaloacetic transaminase, median (IQR) Preoperative Alkaline Phosphatase, median (IQR)	533 (75.4%) 11.3 (8.4–14.9) 1 (0–1) 21 (14–29) 88 (67–112)	53 (44.9%) 8.8 (6.5–11.9) 1 (0–1) 19 (12–30) 76 (53–98)	<0.001 <0.001 <0.001 0.3 <0.001
Intraoperative Variables (%) Emergency Procedure Operative Time (min), median (IQR)	412 62 (46–88)	36 96 (57–150)	<0.001 <0.001
IQR = Interquartile Range, CCY = Cholecystectomy			

### Postoperative outcomes

The outcomes of the multivariate logistic regression are shown in Table 2. Patients who did not undergo concurrent cholecystectomy (CCY) had a longer hospital stay, with an average of 8 days compared to 5 days for those who underwent CCY (*p*<0.01). There was a trend suggesting that patients without CCY were more likely to require a return to the operating room, with 45 cases compared to four cases in the CCY group (*p* = 0.08). However, the difference was not statistically significant.

**Table 2:** Postoperative outcomes for patients undergoing surgical management of gallstone ileus with or without concomitant CCY.

Outcome	No CCY (*n* = 707)	CCY (*n* = 118)	*P*	Estimated Odds Ratio (95% CI)
Total length of hospital stay (days), median (IQR)	8 (5–12)	5 (0–12)	<0.001	**_____________**
30-day Mortality	25 (3.5%)	5 (4.2%)	0.51	1.78 (0.37, 11.6)
Readmissions related to procedure	52 (9.4%)	2 (2.9%)	0.10	0.23 (0.03, 1.03)
Return to Operating Room	45 (6.4%)	4 (3.4%)	0.08	0.33 (0.08, 1.04)
All Surgical Site Infections	102 (14.4%)	12 (10.2%)	0.22	0.62 (0.29, 1.33)
Sepsis	40 (5.7%)	6 (5.1%)	0.74	0.79 (0.21, 3.03)
Intraoperative or Postoperative Transfusions	18 (2.5%)	3 (2.5%)	0.94	0.93 (0.17, 7.6)
Urinary Tract Infection	23 (3.3%)	3 (2.5%)	0.99	(0.22, 4.46)
IQR = Interquartile Range, CI = Confidence Interval, CCY = Cholecystectomy				

The occurrence of surgical site infections (SSIs), readmissions related to the procedure, 30-day mortality, and sepsis were not significantly increased in patients who underwent CCY. Further details regarding the postoperative outcomes are presented in Table 2.

## Discussion

Gallstone ileus accounts for < 5% of small bowel obstruction cases, and the decision to perform concomitant CCY during the index operation has been inconclusive [10], [11], [12], [13], [14]. While gallstone ileus is a rare cause of hospital presentation, it overwhelmingly affects elderly female population, and should be studied further [9,10,13,14]. The findings suggest that omitting concomitant CCY was associated with longer hospital stay and tended towards a higher likelihood of returning to the operating room. Although patient characteristics were accounted for in the statistical models, this finding may reflect strong surgeon selection bias favoring omitting cholecystectomy in patients at higher risk.

The study's primary finding showed that gallstone ileus repair alone, without CCY, was associated with a longer hospital stay. Previous studies have shown that concomitant CCY with gallstone ileus repair should be reserved for younger and healthier populations to prevent longer hospital stays and surgical complications [11], [12], [13], [14], [15], [16], [17], [18]. Chuah et al. described older patients as having a lower physiologic reserve, only tolerating gallstone ileus repair without CCY [4]. Our study supports that concomitant cholecystectomy can be performed in a certain population.

In the no CCY group, patients had an average age of 50 years, whereas, in the CCY group, patients were an average age of 38 years old. Although the patients in the no CCY group were, on average, older than those in the CCY group, their comorbidities were similar. Patients in the no-CCY group had higher preoperative white blood cell counts and alkaline phosphatase levels, both of which may have contributed to the longer length of hospital stay. Similar to other studies, patients in the no CCY and CCY groups were predominantly female [9], [10], [11], [12], [13], [14], [15], [16], [17], [18]. One of the largest series in the literature using the American College of Surgeons National Surgical Quality Improvement Program (ACS-NSQIP) reported no significant difference in mortality rate between CCY and no CCY groups in the surgical management of gallstone ileus. However, the former group experienced a longer surgery and more minor complications [12]. The study's secondary findings included no difference between the CCY and no CCY groups regarding the rates of SSIs, readmissions related to the procedure, 30-day mortality, or sepsis. Previous studies have reported increased mortality rates in patients undergoing concomitant CCY [9], [10], [11], [12], [13], [14], [15], [16], [17], [18]. Per Zuegel et al., gallstone ileus repair with CCY reduces overall mortality compared with gallstone ileus repair alone, as patients in the no CCY group experienced greater postoperative complications leading to mortality [7]. This study found no significant difference between the CCY and no-CCY groups in terms of 30-day mortality.

Gallstone ileus repair has historically been performed using enterolithotomy alone [19]. Mallipedi et al. previously reviewed the NSQIP database for surgical outcomes of gallstone ileus. Their study showed that while there was no difference in post-operative mortality rates between the CCY and no CCY groups, the CCY group experienced longer operations and hospital stay [12]. Using the same database, our study found alternate results as the no CCY group experienced a longer length of hospital stay, eight days, compared with five days in the CCY group. While a shorter hospital stay may be due to a younger patient population, it proposes a different finding to be considered in patient management [9], [10], [11], [12], [13], [14], [15], [16], [17], [18]. In 2022, Vera-Mansilla et al. proposed that enterotomy alone was sufficient and that CCY may be unnecessary [20]. Our study showed that concomitant CCY was not associated with worse outcomes and may be a viable option for younger patients without significant comorbidities.

The long-term comparability and clinical relevance of gallstone ileus repair with CCY versus gallstone ileus repair without CCY are also relevant considerations. Several studies have indicated that gallstone ileus repair with concomitant cholecystectomy provides better long-term outcomes by reducing the risk of recurrent gallstone-related complications, such as recurrent ileus and cholangitis [3,7]. These findings suggest that cholecystectomy during gallstone ileus repair contributes to preventing future gallstone-related problems. However, other studies have not favored CCY for various reasons [9], [10], [11], [12], [13], [14], [15], [16], [17], [18]. Though this study has demonstrated decreased length of hospital stay in patients with concomitant CCY, individual patient factors, such as overall health status and surgical risks, as well as the presence of concurrent gallbladder pathology, should be considered when determining the appropriateness of performing concomitant cholecystectomy. No randomized controlled trials explore long-term comparability; otherwise, further studies are needed to evaluate concomitant CCY at the time of gallstone ileus repair.

### Limitations

While the study proposes new and supports other findings regarding concomitant CCY during gallstone ileus repair, there were limitations. Given the study's retrospective nature, it is unclear how surgeon-specific judgment and experience affected the decision to perform CCY during gallstone ileus repair. As previously discussed, surgeons may not perform concomitant CCY in patients with poor outcomes. However, this is only a theory since we could not access operative notes. Additionally, because the study relied on a database with preset variables, it is possible that important measures specific to gallstone ileus repair and CCY were omitted. For example, emergent hepatobiliary procedures are often associated with increased alkaline phosphatase levels and white blood cell counts. However, using the preset database, we could not determine whether patients with elevated alkaline phosphatase levels and white blood cell counts were the same as patients with emergent procedures and increased lengths of hospital stay. Further studies are required to evaluate this heavily debated surgical topic fully.

## Conclusion

Surgical management of gallstone ileus with or without cholecystectomy has similar short-term postoperative outcomes. Our findings suggest that omitting concomitant cholecystectomy was associated with a longer hospital stay and tended to be more likely to return to the operating room. No CCY was associated with increased rates of SSIs, readmissions related to the procedure, 30-day mortality, or sepsis.

### Funding sources

No specific grants were received from the public, commercial, or not-for-profit sectors to fund this research.

### Data availability

The authors of this study utilized the American College of Surgeons (ACS) National Surgical Quality Improvement Program (NSQIP) database, which is exclusively accessible to ACS members. However, owing to restrictions on data redistribution, the authors could not provide access to the specific data used in the study.

## Declaration of Competing Interest

The authors declare that they have no known competing financial interests or personal relationships that could have appeared to influence the work reported in this paper.
